# Evaluation of ganglion cell complex in patients taking hydroxychloroquine

**DOI:** 10.1186/s12886-025-04247-w

**Published:** 2025-09-09

**Authors:** Prativa Acharya, Pragati Gautam Adhikari, Ranju Kharel Sitaula, Madhu Thapa

**Affiliations:** Department of Ophthalmology, Institute of Medicine, Tribhuvan University, B.P Koirala Lions Centre For Ophthalmic Studies, Kathmandu, Nepal

**Keywords:** Focal loss Volume(FLV), Ganglion cell complex (GCC), Global loss Volume(GLV), Hydroxychloroquine (HCQ), Spectral domain optical coherence Tomography(SD-OCT)

## Abstract

**Background:**

To evaluate the ganglion cell complex thickness in patients taking oral hydroxychloroquine.

**Methods:**

In this hospital-based, cross-sectional, non-interventional, comparative study, 87 eyes of 87 patients taking hydroxychloroquine were recruited. All the patients underwent complete ophthalmological evaluation along with dilated fundus examination. Patients were divided into two groups based on the duration of hydroxychloroquine intake: Group 1 (62 patients having duration of hydroxychloroquine intake < 5 years) and Group 2 (25 patients having duration of hydroxychloroquine intake ≥ 5 years). Age and gender-matched healthy volunteers with normal ocular findings were taken as controls. Average, superior, inferior GCC thickness, focal loss volume, and global loss volume were measured by RTvue XR Avanti SD-OCT.

**Results:**

The average, superior, and inferior GCC thicknesses were significantly reduced in both Group 1 (< 5 years) and Group 2 (≥ 5 years) as compared to controls (*p* < 0.001). In Group 1, mean GCC values were 94.70 ± 6.34 μm (average), 94.43 ± 6.28 μm (superior), and 94.74 ± 6.81 μm (inferior), while the corresponding values in controls were 99.79 ± 4.61 μm, 99.38 ± 4.63 μm, and 99.97 ± 4.61 μm. Likewise, FLV and GLV in Group 1 were 1.76 ± 2.5% and 4.07 ± 3.27%, significantly higher than in controls (0.72 ± 0.45% and 1.39 ± 1.1%, respectively; *p* = 0.002 for FLV, *p* = 0.001 for GLV). Similarly, in Group 2, mean GCC thicknesses were 92.70 ± 6.39 μm (average), 92.44 ± 5.92 μm (superior), and 93.32 ± 7.25 μm (inferior), all significantly lower than in controls (*p* < 0.001). While GLV was significantly elevated in Group 2 (4.46 ± 4.42%; *p*-0.003), the difference in FLV (1.23 ± 1.12%) was not statistically significant compared to controls (*p*-0.077).

**Conclusion:**

The Ganglion Cell Complex thickness was significantly thinner in patients taking hydroxychloroquine along with elevated Focal Loss Volume % and Global Loss Volume %. However, no statistically significant correlation was observed between GCC thickness and duration of hydroxychloroquine use.

## Background

Hydroxychloroquine is a 4-aminoquinoline derivative widely prescribed for treating several rheumatologic and dermatologic disorders, including systemic lupus erythematosus and rheumatoid arthritis [1–3]. Retinal toxicity is the major and potentially most serious irreversible side effect of this treatment, manifesting as bull’s eye maculopathy, irreversible loss of visual acuity, color vision defects, and central scotoma [4–7]. The overall prevalence of hydroxychloroquine retinopathy is 7.5%,which varied with daily consumption (odds ratio, 5.67; 95% CI, 4.14–7.79 for > 5.0 mg/kg) and with duration of use (odds ratio, 3.22; 95% CI, 2.20–4.70 for > 10 years) [8], indicating the strong dose- and duration-dependent associations.

**Fig. 1:**
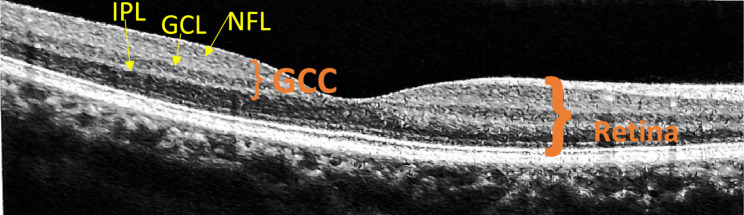
OCT image of healthy 35Y/F showing the segmentation of GCC layer comprised of the NFL, GCL, and IPL compared to the segmentation of the entire retina [taken with consent by RTvue XR avanti], Source: Authors

The ganglion cell complex consists of three layers of retina: the nerve fiber layer (NFL), the ganglion cell layer (GCL), and the inner plexiform layer (IPL) [9] (Fig. 1). As a type of 2nd order neuron, GCC plays a vital role in visual processing [10]. It is usually a single layer in the peripheral retina; within the macula, it is multilayered and has the highest concentration of ganglion cells [11]. In the initial stages of toxicity, changes are seen in retinal ganglion cells in the form of membranous cytoplasmic bodies followed by cellular degeneration [12]. In this stage, the toxicity is difficult to diagnose, as fundus examination may appear normal. At later stages, it leads to the degeneration of photoreceptors and retinal pigment epithelium degeneration by binding to melanin resulting in functional loss in the visual field, decreased visual acuity, and impairment of color vision. Since the structural and functional damage at later stages is irreversible, an early screening for hydroxychloroquine retinopathy becomes crucial. Also, baseline screening is recommended before starting HCQ treatment to rule out pre-existing maculopathy [13]. OCT is a non-invasive retinal imaging technique that produces cross-sectional images of different layers of the retina and used for quantitative analysis of retinal morphology (Fig. 1). The ability of OCT to visualize the retinal tissue with ultra-high clarity in a fraction of seconds and have ushered in a new era of retinal pathology documentation and diagnostic imaging [14].

RTVue XR Avanti SD-OCT provides GCC parameters like average, superior, and inferior GCC thickness along with global loss volume (GLV%) and focal loss volume (FLV%) of ganglion cell complex. Spectral-domain optical coherence tomography shows localized thinning of retinal layers in the parafoveal zone and may reveal retinal toxicity at an earlier stage before visual field loss or any retinal signs appear [15–17].

## Patients and methods

Eighty-seven patients taking HCQ and eighty-seven age-matched healthy subjects were enrolled in this hospital-based, cross-sectional, and comparative study. Approval from the Institutional Review Committee of Institute of Medicine was taken [IRC no: 233 (6-11) E_2_(080/081)]. Informed consent was obtained from all the patients. The inclusion criteria for patients included using HCQ for at least 3 months and above 18 years or older. The control subjects were healthy individuals with normal ocular and systemic findings. Subjects having any kind of macular pathology, optic neuropathy, high refractive error (+/- 4.00D), media opacities, or history of intraocular surgery were excluded from the study. Information about systemic disease diagnosis, dosage, and duration of HCQ intake was noted. All the patients underwent a complete ophthalmic examination, which included visual acuity testing, refraction, air puff tonometry, and dilated fundus evaluation with + 20D or + 90D. The patients taking hydroxychloroquine were divided into subgroups based on the duration: duration less than 5 years (Group 1) and duration more than 5 years (Group 2).

###  GCC Analysis

**Fig. 2:**
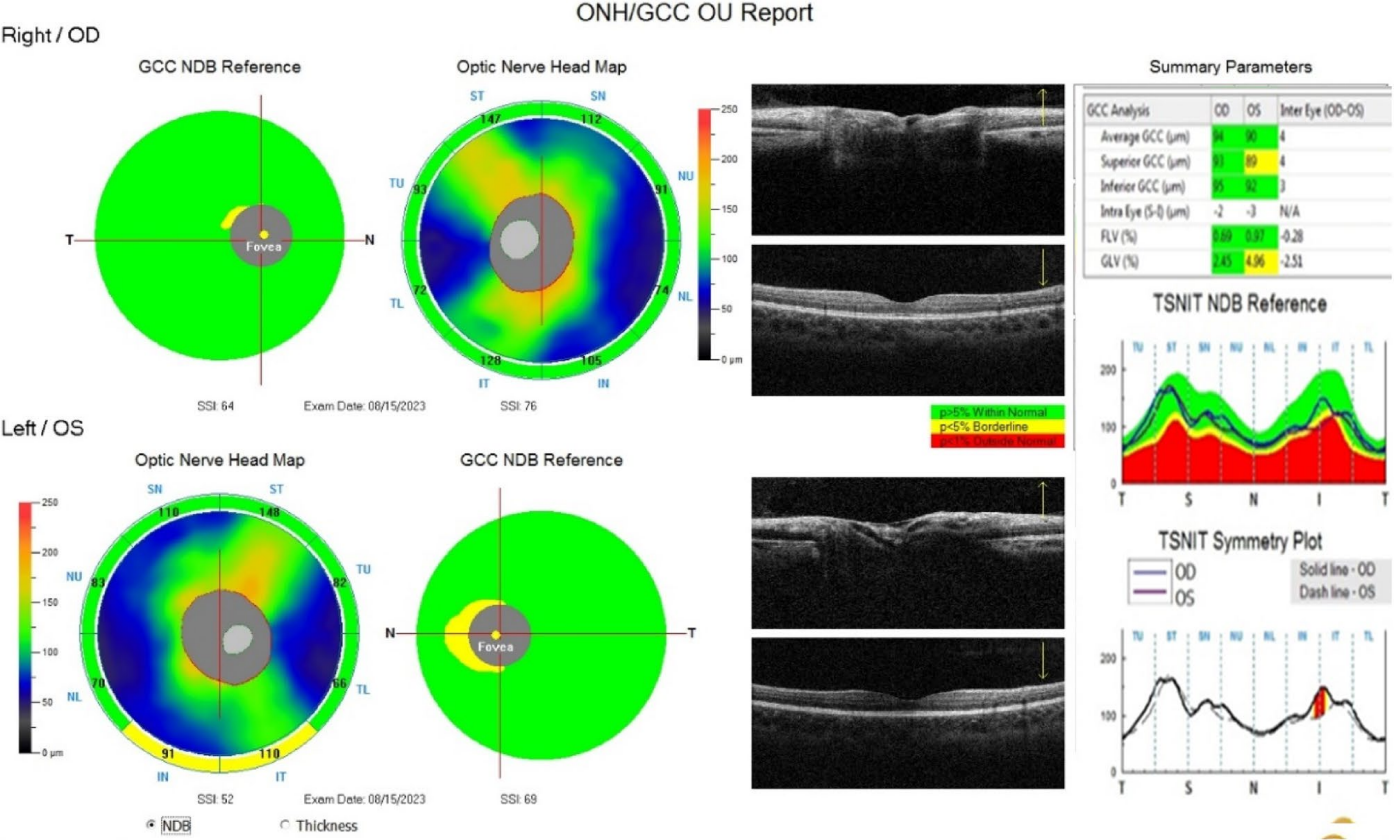
Figure showing the both-eyes GCC analysis report of an 18-year-old female diagnosed with SLE taking oral hydroxychloroquine for 1 year. The optic nerve head analysis map shows normal cup to disc ratio. The GCC analysis shows thinning of GCC (LE > RE), Source: Authors

Ganglion cell analysis was done by RTVue XR Avanti SD-OCT (Optovue Inc., Fremont, CA). GCC parameters were assessed by the MM7 protocols, which use one horizontal line with a 7 mm scan length (934 A-scans) and 15 vertical lines with a 7 mm scan length and 0.5 mm interval (800 A-scans) centered 1 mm temporal to the fovea [18]. The GCC thickness was measured between the internal limiting membrane and inner plexiform layer. The focal loss volume is the total sum of significant GCC loss (in volume) expressed as a percentage of map area providing the significant tissue loss for the volume. Global Loss Volume is the average amount of GCC loss over the entire GCC map. GLV is the sum of the pixels where the fractional deviation map value is < 0, divided by the total map area to give a percent loss of GCC thickness. (Fig. 2).

### Statistical analysis

Collected data was checked, reviewed, and organized for completeness and accuracy, and it was entered into the Statistical Package for Social Science (SPSS version 26, IBM Corp., Armonk, N.Y., USA) for analysis. Kolmogorov-Smirnov test was used to determine whether the parameters are normally distributed. An average from both eyes was taken for statistical analysis. Comparisons of continuous variables between the groups were performed using independent sample t-tests. Pearson’s correlation was used to correlate the variables. A value of *p* < 0.05 was considered statistically significant.

## Results

### Demographic and clinical characteristics 

The best corrected visual acuity was 20/20 in all the cases. The anterior segment and fundus findings were normal. The overall mean age for patients taking hydroxychloroquine was 40.9 years. Patients with HCQ consumption duration less than 5 years (Group 1) had a minimum age of 18 years and a maximum age of 69 years (Mean = 41.4 ± 15 years). The mean age for patients having duration of HCQ consumption of more than 5 years (Group 2) was 39.7 ± 12 years while the minimum age was 20 years and the maximum age was 66 years.

The male: female ratio was 10:77. The patients taking hydroxychloroquine for less than 5 years were 62, comprising 71.2% of the total cases (4 males and 58 females), while the patients taking hydroxychloroquine for more than 5 years were 25, comprising 28.8% of total cases (6 males and 19 females). The mean dosage in patients having a duration of HCQ intake of less than 5 years was 216.12 mg/day while it was 228 mg/day in patients having duration of HCQ consumption of more than 5 years. The overall mean dosage in all the patients enrolled in study was 219.5 mg/day. The mean duration of HCQ intake was 3.44 years. The maximum duration of HCQ intake was 15 years, and the minimum duration was found to be 4 months.

**Fig. 3:**
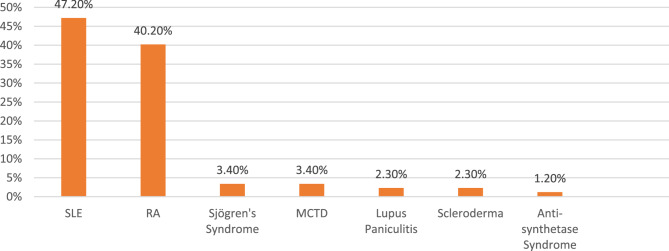
Bar graph showing the systemic disease diagnosis of patients taking HCQ. (Source: Authors)

Out of 87 patients enrolled in the study, most were diagnosed with SLE, followed by RA (Fig. 3).

### Ganglion cell complex measurements

The mean average, superior and inferior ganglion cell complex thickness, along with mean FLV and GLV of Group 1,Group 2, Controls, patient with SLE and patients with RA is shown in the table below. The average, superior and inferior GCC thickness were significantly lower in both Group 1 and Group 2 as compared to controls (*p* < 0.001) (Table 1).

**Table 1:** Comparison of ganglion cell complex parameters among group 1, group 2, control, and Disease-Specific subgroups

GCC Parameters	Group 1 (< 5 yrs HCQ)	Group 2 (≥ 5 yrs HCQ)	Control	*p*¹	*p*²	*p*³	SLE	RA	*p*⁴
Average (µm)	94.70 ± 6.34	92.70 ± 6.39	99.79 ± 4.61	0.224	< 0.001**	< 0.001**	94.52 ± 5.3	93.08 ± 6.19	0.281
Superior (µm)	94.43 ± 6.28	92.44 ± 5.92	99.38 ± 4.63	0.202	< 0.001**	< 0.001**	93.79 ± 5.5	92.81 ± 5.79	0.457
Inferior (µm)	94.74 ± 6.81	93.32 ± 7.25	99.97 ± 4.61	0.460	< 0.001**	< 0.001**	94.80 ± 5.41	93.41 ± 6.76	0.323
FLV (%)	1.76 ± 2.5	1.23 ± 1.12	0.72 ± 0.45	0.302	0.002*	0.077	1.35 ± 1.46	1.78 ± 2.75	0.386
GLV (%)	4.07 ± 3.27	4.46 ± 4.42	1.39 ± 1.1	0.168	0.001*	0.003*	3.78 ± 4.08	4.61 ± 4.00	0.378

*GC, *Ganglion Cell Comple,* FLV* Focal Loss Volume,* GLV* Global Loss Volume * HCQ *Hydroxychloroquine,* RA *Rheumatoid Arthritis,SLE Systemic Lupus Erythematosus

Data are expressed as mean ± SD

*p < 0.05, **p < 0.001. (*p* is statistically significant at *p*<0.05)

*p*¹ = Comparison between Group 1 and Group 2 (Independent sample t-test)

*p*² = Comparison between Group 1 and Control(Independent sample t-test)

*p*³ = Comparison between Group 2 and Control(Independent sample t-test)

*p*⁴ = Comparison between SLE and RA(Independent sample t-test)

### Correlation between GCC parameters and duration of hydroxychloroquine

In patients taking hydroxychloroquine for less than five years, all the GCC parameters except average GCC and GLV showed negative correlations which were statistically insignificant. In patients taking hydroxychloroquine for more than five years, all the parameters except for GLV showed a negative correlation which was not statistically significant (Table 2).

**Table 2:** Correlation between GCC parameters and duration of hydroxychloroquine

GCC Parameters	Group 1 (< 5 yrs HCQ)		Group 2 (≥ 5 yrs HCQ)	
	*r*	*p* ^1^	*r*	*p* ^1^
Average(µm)	0.011	0.930	−0.036	0.865
Superior(µm)	−0.16	0.899	−0.11	0.957
Inferior(µm)	−0.47	0.718	−0.102	0.628
FLV%	−0.13	0.920	−0.049	0.817
GLV%	0.45	0.726	0.039	0.855

*GCC* Ganglion Cell Complex, *FLV* Focal Loss Volume, *GLV* Global Loss Volume, *HCQ* Hydroxychloroquine

*r* = Pearson’s correlation coefficient, *p*¹ = *p*-value from Pearson’s correlation test

A *p*-value < 0.05 was considered statistically significant

## Discussion

In this analytical, cross-sectional, comparative, and hospital-based study, we compared the Ganglion Cell Complex (GCC) thickness between patients taking hydroxychloroquine and healthy individuals by RTvue SD-OCT. In addition to this, the correlation between the duration of hydroxychloroquine intake and GCC parameters was analyzed. Hydroxychloroquine’s use for various rheumatologic and dermatological conditions is steadily increasing daily [19]. Various studies have reported that the first pathological changes occur in retinal ganglion cells [12, 20]. As the drug accumulates in the cytoplasm of these cells, it can cause cell shrinkage and irregular cell body formation, eventually leading to ganglion cell degeneration. In the initial phases, hydroxychloroquine treatment may not manifest significant side effects. However, as the condition progresses, patients may develop characteristic symptoms such as bull’s-eye maculopathy, paracentral scotoma, reduced color perception, and central vision impairment. It’s important to note that in advanced stages, these effects can become irreversible and persist even after discontinuing hydroxychloroquine therapy [21]. Since ganglion cells are affected in the early stages of the toxicity, assessment of ganglion cell thickness in early courses of treatment may be helpful in detection of hydroxychloroquine toxicity before any kind of changes in the fundus are seen. SD-OCT technology offers high-resolution, cross-sectional imaging of the retina, making it a suitable objective screening tool for HCQ retinal toxicity. Utilizing such advanced technology may enhance early detection and monitoring of HCQ-induced retinal changes, potentially mitigating irreversible visual impairment [4].

In our study, among the 87 patients receiving hydroxychloroquine, the majority of 77 were female, yielding a sex ratio of 7:1. This aligns with findings from Kvien et al., who noted a higher prevalence of rheumatic diseases, including rheumatoid arthritis (RA), among females, with a prevalence ratio of 5:1 compared to males [22].

In our study, the mean dosage of hydroxychloroquine was found to be 216.12 ± 45.06 mg/day in group 1, while it was 228 ± 61.37 mg/day in group 2, which was almost similar to that of a study done by Kim et al., which included 132 patients with the mean dosages of 243.6 ± 67.5 mg/day [23]. The mean duration of hydroxychloroquine was 3.4 years. The predominant diagnosis among patients receiving hydroxychloroquine was systemic lupus erythematosus (47.2%), followed by rheumatoid arthritis (40.2%) and Sjögren’s syndrome (3.4%). This distribution differs somewhat from the observations of Jokar et al., whose study included 12,626 patients and reported rheumatoid arthritis (47.3%) to be the most prevalent condition, followed by spondyloarthropathies (17.23%), SLE (8.1%), gout (7.84%), and vasculitis (6.84%) [24].

In Group 1, the Ganglion Cell Complex (GCC) parameters were as follows: Average GCC thickness was 94.70 ± 6.34 μm, superior GCC thickness was 94.43 ± 6.28 μm, and inferior GCC thickness was 94.74 ± 6.81 μm. Additionally, Focal Loss Volume (FLV) was 1.76 ± 2.5%, and Global Loss Volume (GLV) was 4.07 ± 3.27%. In Group 2, the corresponding values were an average GCC thickness of 92.70 ± 6.39 μm, a superior GCC thickness of 92.44 ± 5.92 μm, and an inferior GCC thickness of 93.32 ± 7.25 μm. FLV and GLV were measured at 1.23 ± 1.12% and 4.46 ± 4.42%, respectively. No significant difference was observed between the superior and inferior GCC thicknesses. We also didn’t find a statistically significant difference between the GCC parameters of Group 1 and Group 2. Comparing these findings to the study by Kan et al., wherein 90 patients (mean age = 48.3 ± 9.2 years) taking hydroxychloroquine (an average of 6.5 mg/kg/day) exhibited average, superior, and inferior GCC thicknesses of 82.2 ± 5.5 μm, 82.7 ± 5.7 μm, and 82.4 ± 4.7 μm, respectively, differences in GCC thickness were evident [21]. These differences in GCC thickness may be attributed to using different Spectral Domain Optical Coherence Tomography (SD-OCT) devices across both studies, emphasizing the impact of technological differences on GCC thickness measurements.

**Fig. 4:**
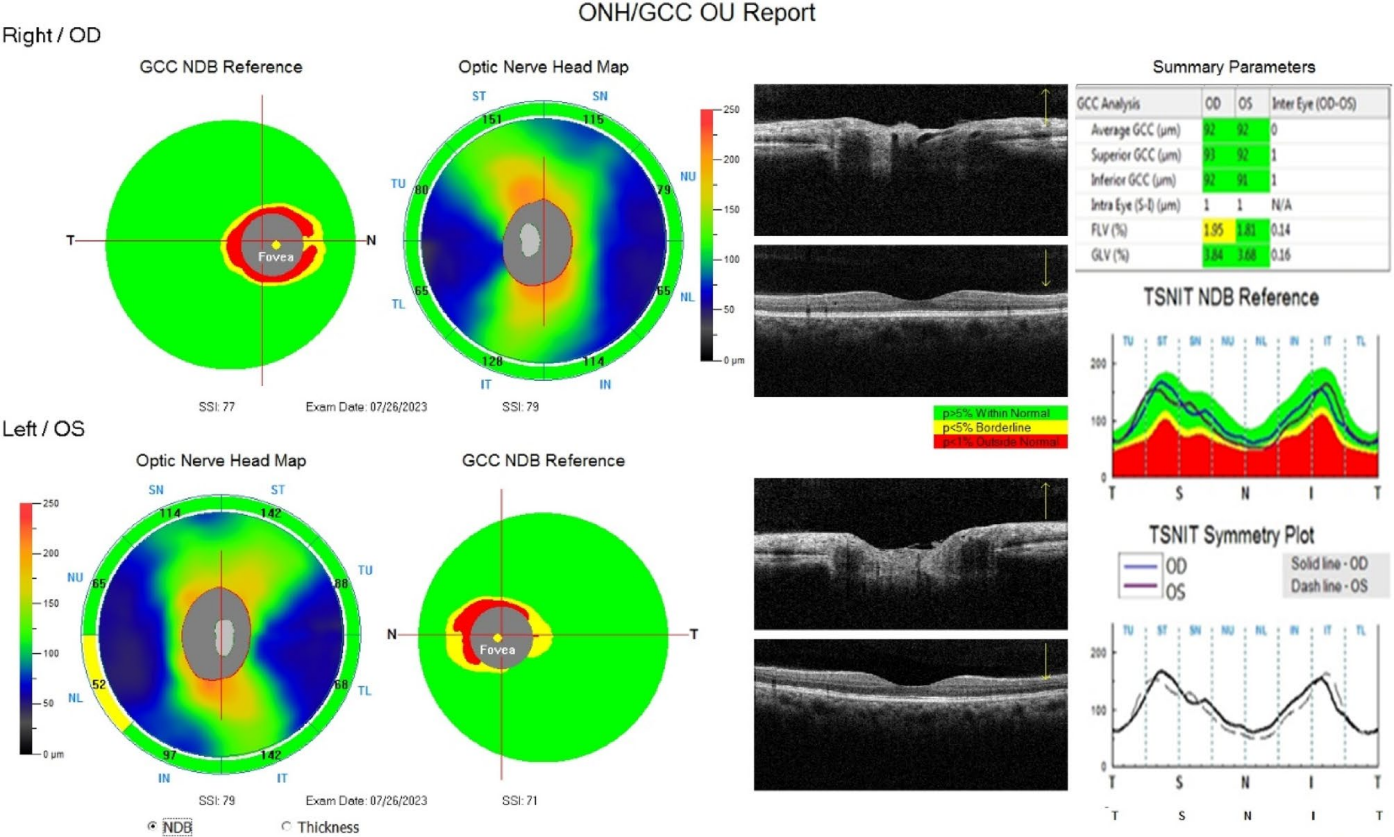
Figure showing the both-eyes GCC analysis report of a 40-year-old female diagnosed with RA taking oral hydroxychloroquine for 7 years. The optic nerve head map shows normal cup-to-disc ratio. The GCC analysis shows thinning of GCC. Source: Authors

The reduction of GCC thickness in both Group 1 and Group 2 as compared to the control indicates the thinning of the average, superior, and inferior GCC in patients taking hydroxychloroquine (Fig. 4). This thinning might be due to the accumulation of the drug in the cytoplasm of ganglion cells, leading to cell shrinkage and degeneration [12]. The FLV% quantifies the amount of significant GCC loss, and the increased amount of focal loss volume in Group 1 suggests that there may be localized retinal damage in patients taking hydroxychloroquine in the early course of HCQ therapy. The GLV % quantifies the average amount of GCC loss over the GCC map. A significantly higher GLV% in Group 2 indicates that there may be widespread damage of retinal ganglion cells along with localized damage in patients taking HCQ for a longer time.

The study by Agcayazi et al. including 40 patients (mean age 48.4 ± 14.4 years), reported thinner GCC thickness in all quadrants was significant in patients compared to healthy controls (p- 0.017, p-0.001, p-0.019, p −0.001 in superonasal, superotemporal, inferonasal, and inferotemporal) [25]. This aligns with our findings, which indicates a consistent pattern of GCC thinning in patients taking hydroxychloroquine. Similarly, Bulut et al. observed thinner GCC in 46 patients (mean age 53.6 ± 8.1 years) taking HCQ, particularly at an early stage [26]. Together, these results highlight how HCQ may affect GCC thickness even during the early stages of treatment. Hence, early detection of hydroxychloroquine toxicity also provides guidelines for manipulating the dosage and duration of hydroxychloroquine, which in turn reduces the prevalence of hydroxychloroquine toxicity.

Sallam et al. reported decreased OCT measurements in 30 patients taking hydroxychloroquine as compared to the control group, with all the measurements showing statistical significance (*p* < 0.001) [27]. This highlights the repeatability of these findings across many investigations and further validates our observations of GCC thinning in patients treated with HCQ.

Additionally, we did not observe any statistically significant difference in GCC parameters while comparing our two predominant diagnoses (SLE and RA). However, patients with RA had slightly elevated FLV and GLV values, suggesting a varied pattern of retinal involvement.

In our study, we observed weak negative correlations between HCQ intake duration and GCC parameters. In Group 1 (< 5 years), superior GCC, inferior GCC, and FLV showed weak negative correlations (p −0.899, 0.718, 0.920).Similarly, in group 2, similar correlations were observed in average GCC, superior GCC, inferior GCC, and FLV with p-values of 0.865, 0.957, 0.628, and 0.817. In a study by Lee et al., which included 130 patients (mean age = 41.3 years) with a mean dosage of 292.5 mg/day, the average and minimum Ganglion Cell Complex-Inner Plexiform Layer (GCC-IPL) thicknesses did not exhibit a significant correlation with the duration of hydroxychloroquine intake among patients without retinopathy (p-values of 0.125 and 0.033, respectively) [28]. This correlation suggests that with the increase in the duration of hydroxychloroquine intake, there is a decrease in GCC thickness. However, the correlations were not statistically significant.

When considered collectively, the consistency of these findings across several investigations emphasizes the potential value of GCC measurements as a sensitive indicator of retinal changes linked to HCQ use and emphasizes the significance of tracking GCC thickness in patients undergoing hydroxychloroquine therapy.

This study offers significant insights into the retinal changes due to HCQ use. We evaluated both early and prolonged exposure effects by stratifying patients according to treatment duration (< 5 years vs. ≥5 years), which are frequently underreported. Alongside structural GCC measures, we incorporated FLV and GLV as functional indicators of localized and diffuse ganglion cell degeneration. The pattern of elevated FLV in Group 1 and elevation of GLV in Group 2 might suggest the progression from focal to more generalized retinal damage. Furthermore, we also did subgroup analysis by diagnosis (SLE and RA), offering insights into retinal involvement in different autoimmune conditions. Also, this study is among the few studies to investigate HCQ toxicity among the Nepalese population, providing information from the region that is not well represented in literature. Furthermore, GCC analysis was performed using RTvue SD-OCT, allowing for comparison across technologies.

The inability to conduct a longitudinal follow-up in our study limits the ability to assess changes in GCC thickness over time or establish a causal relationship between HCQ use and retinal thinning. Another drawback of our study was that GCC thickness was measured only superiorly and inferiorly; measuring GCC thickness in all four quadrants would have provided more detailed information.

## Conclusion

In conclusion, our study demonstrates significant thinning of the ganglion cell complex in patients taking oral HCQ. This difference, with notable correlations, was observed in both Group 1 (duration < 5 years) and Group 2 (duration > 5 years) as compared to controls. Elevated FLV and GLV in patients suggest potential retinal structural alterations associated with HCQ use. Although the duration of HCQ intake did not show a statistically significant correlation with GCC thickness, FLV, or GLV, the overall finding highlights the importance of early monitoring.

## Data Availability

Data is provided in supplementary files in Excel file format.

## Abbreviations

OCT: Optical Coherence Tomography
GCC: Ganglion Cell Complex
HCQ: Hydroxychloroquine
FLV: Focal Loss Volume
GLV: Global Loss Volume
